# Anthropometric Characteristics of Metacarpal Bones in the Mexican Population: A Retrospective Analysis

**DOI:** 10.7759/cureus.82659

**Published:** 2025-04-20

**Authors:** Humberto Osnaya-Moreno, Yusef Jiménez-Murat, Victor Hugo Garzón Ortega, Alejandro Hernandes Moreno, Jesús C Ceballos Villalva

**Affiliations:** 1 División de Cirugía Plástica y Reconstructiva, Hospital Medica Sur, Mexico City, MEX; 2 División de Cirugía Plástica y Reconstructiva, Hospital General Dr. Manuel Gea González, Mexico City, MEX; 3 Departamento de Cirugía General, Christus Muguerza Hospital de Alta Especialidad, Monterrey, MEX; 4 Servicio de Cirugía Plástica, Hospital General Adolfo López Mateos, Instituto de Seguridad y Servicios Sociales de los Trabajadores del Estado (ISSSTE), Mexico City, MEX

**Keywords:** anthropometry, bone anthropometry, bone measurements, hand anatomy, metacarpal bones, plastic and reconstructive surgery, skeletal variation

## Abstract

Introduction: In Mexico, there is limited evidence regarding the anthropometric characteristics of metacarpal bones in the Mexican population. Understanding these characteristics is crucial for improving surgical interventions and ensuring optimal hand functionality. Metacarpal bones exhibit variations in length and width based on sex and geographic region of origin or residence. This study aims to describe the anthropometric characteristics of metacarpal bones in Mexican patients.

Materials and Methods: A retrospective, observational, descriptive, and cross-sectional study was conducted from January 1 to June 30, 2019. The dimensions of the metacarpal bones (axial length, proximal epiphysis width, distal epiphysis width, and diaphysis width) were measured using the Synapse Mobility Login system (Fujifilm Medical Systems, Tokyo, Japan). Patient age and sex were recorded, and statistical analysis was performed using SPSS software, version 25.0 (IBM Corp., Armonk, NY).

Results: A total of 400 cases were included, comprising 215 men (49.5 ± 15.86 years) and 185 women (53.2 ± 14.36 years). Overall, men exhibited larger dimensions across all five metacarpal bones, particularly in proximal epiphysis width and axial length, except for the third metacarpal. Normality tests (Shapiro-Wilk and Kolmogorov-Smirnov) indicated non-normal distributions for all measurements. Non-parametric tests (Mann-Whitney and Wilcoxon W) revealed no statistically significant differences between sexes.

Conclusion: No significant differences in mean metacarpal measurements were found between the sexes. The lack of normal data distribution and statistical significance underscores the need to expand measurements to other hand bone structures and include more representative samples to enhance the accuracy of future studies.

## Introduction

The measurements of different parts of the human body, or anthropometry, have been conducted since the dawn of history. These measurements have been useful for medical and artistic purposes and are now widely applied to studying relationships with physical performance, ergonomics, tool design, safety equipment, personal protection, and more. Different minimal findings in bones can be related to sexual characteristics, age, height, racial affiliation, or other factors. In general, age and sex provide the greatest differentiation of these characteristics [1].

The word "metacarpus" is derived from the Greek meta, meaning "beyond," and carpus, meaning "wrist." The metacarpus consists of five cylindrical bones classified as long bones due to the presence of a medullary canal. These bones feature a shaft or diaphysis that widens at its proximal end (carpal region) to articulate with the corresponding carpal bones and proximally with the phalanges. The diaphysis extends toward the distal end, known as the digital region or head, with rounded articular surfaces that connect to the phalanges. When the fingers are in full flexion, these articular surfaces can be palpated at the metacarpophalangeal joints [2,3].

Differentiating the characterization of each metacarpal bone, as in forensic medicine, supports human identification and sex estimation. In reconstructive surgeries, unique characteristics and measurement trends help plan surgical procedures and determine the appropriate approach [1]. In forensic medicine, characterizing these bones aids in determining sex when long bones are absent or partially destroyed [4-6]. A hospital-based study in Egypt used radiographic studies to measure the dimensions of the metacarpals and phalanges in the population, concluding that the lengths of the metacarpals and phalanges, particularly the first distal phalanx, proximal phalanx, and the 3rd and 4th metacarpals, could be used for sex determination [4].

Various studies have shown variability in bone measurements, with a consistent finding being the greater length of the second metacarpal compared to the others, and the third metacarpal always being longer than the fourth [7-9]. Although there is no recent study on measurements in the Mexican population, studies have been conducted on foreign populations. In 2011, Eshak et al. determined the lengths of the metacarpals in young Egyptians using computed tomography, studying a group of 60 men and 52 women. The difference in metacarpal length between sexes was approximately 5 mm longer in males [10]. Similarly, Khanpetch et al. studied skeletons from the Thai population (295 skeletons) and established clear differences in bone diameter, epiphyseal width, and base width according to sex. Both studies suggest that all five metacarpal bones are useful for determining sex in their study populations [11].

Accurately evaluating the anatomy of the metacarpus can be challenging, but remains important. However, there is an absence of osteometric guides for metacarpals, despite their frequent measurement and use. The closest reference is the proposal by Scheuer and Elkington for sex determination in forensic medicine. This evaluation involved measurements of interarticular distance, head and base width, and mid-shaft diameter [12,13].

Other studies have used population measurements to support predictive algorithms. For example, Mennatallah et al. conducted the first study on the Egyptian population that used medullary and cortical measurements of the first and second metacarpals through tomography in 3D models. This study produced precision equations for their population, useful for determining sex when commonly used bones are absent or when mixed bones are destroyed, such as in cases of mass disasters in their population [14].

In Mexico, there is limited evidence regarding the anthropometric characteristics of the population. It is known that metacarpals can vary in length, width, and characteristics depending on sex and geographic origin or residence. This study aims to describe the anthropometric characteristics of metacarpal bones in a Mexican population using radiographic measurements and to evaluate potential sex-based differences in patients treated in the Plastic Surgery Department of the “Dr. Manuel Gea González” Hospital from January 1, 2019, to June 30, 2019.

## Materials and methods

An observational, descriptive, retrospective, and cross-sectional study was conducted using the radiological archive of patients treated by the Division of Plastic and Reconstructive Surgery at the General Hospital "Dr. Manuel Gea González" between January 1, 2019, and June 30, 2019. All procedures adopted in this study adhered to the ethical standards outlined in the Declaration of Helsinki.

The study included patients over 18 years of age with anteroposterior hand radiographs and excluded those with bone tissue loss, previous structural hand injuries, or hand surgeries. For each patient, demographic data (age and sex) and radiographic measurements (axial length, proximal epiphysis width, distal epiphysis width, and diaphysis width) were recorded. All measurements were performed using the Synapse Mobility Login imaging viewer (Fujifilm Medical Systems, Tokyo, Japan), ensuring precision to the nearest millimeter [15].

Descriptive statistics were calculated for each metacarpal measurement, and the normal distribution of the data was assessed using the Shapiro-Wilk and Kolmogorov-Smirnov tests. Non-parametric tests (Mann-Whitney U and Wilcoxon W) were used for group comparisons due to the non-normal distribution of the data. All analyses were performed using SPSS software, version 25.0 for Windows (IBM Corp., Armonk, NY) [16].

## Results

A total of 400 cases were analyzed, comprising 215 men (49.5 ± 15.86 years) and 185 women (53.2 ± 14.36 years). The anthropometric measurements of the metacarpal bones, including axial length (Longaxial), proximal epiphysis width (Ancepiprox), distal epiphysis width (Anchepidis), and diaphysis width (Anchdiaf), were recorded for all five metacarpals. The general features commonly known for each metacarpal are described in Table 1.

**Table 1:** Characteristics of the metacarpal bones References [2-5].

	1st Metacarpal	2nd Metacarpal	3rd Metacarpal	4th Metacarpal	5th Metacarpal
Structure	Closely articulated with other metacarpals. The body is shorter, wider, and flatter than the others.	The base has a deep notch accommodating the trapezoid. Includes an articular surface for the trapezium.	Projection from the trapezoid and capitate bones.	The base articulates with the capitate and hamate bones.	Articular facet for the fourth metacarpal. The base includes an articulation area for the hamate bone.
Bone Characteristics	The only metacarpal with a saddle joint.	Longest of the metacarpals.	Contains a styloid process. Larger than the fourth metacarpal.	The elongated structure is commonly compared to the third metacarpal.	It's the smallest of the set.

First metacarpal

The measurements for the first metacarpal are summarized in Table 2. Men exhibited slightly larger axial lengths (mean: 48.17 mm) compared to women (mean: 47.76 mm), but the difference was not statistically significant. Similarly, proximal epiphysis width was greater in men (mean: 14.54 mm) than in women (mean: 14.75 mm), though the difference was minimal. Distal epiphysis width and diaphysis width also showed no significant differences between sexes.

**Table 2:** Anthropometry of the first metacarpal

Measurement (mm)	Sex	Mean	95% Confidence Interval	Variance	Standard Deviation	Minimum	Maximum	Range
Axial Length	F	48.1678	47.5211 - 48.8144	19.875	4.4581	36.69	58.31	21.62
	M	47.756	47.5211 - 48.8144	19.875	4.4581	36.79	58.09	21.30
Proximal Epiphysis Width	F	14.7455	14.2658 - 15.2253	10.939	3.3074	9.30	23.65	14.35
	M	14.5407	14.2658 - 15.2253	11.041	3.32273	9.32	24.40	15.08
Distal Epiphysis Width	F	15.19	14.8497 - 15.5326	5.542	2.35408	9.88	21.75	11.87
	M	15.2327	14.9164 - 15.549	5.538	2.35324	11.49	21.64	10.15
Diaphysis Width	F	10.9795	10.3189 - 11.6401	20.74	4.55407	0.63	20.56	19.93
	M	11.0973	10.523 - 11.6717	18.257	4.27282	0.63	21.43	20.80

Second metacarpal

The results for the second metacarpal are presented in Table 3. The axial length was nearly identical between men (mean: 69.40 mm) and women (mean: 69.42 mm). Proximal epiphysis width was slightly larger in women (mean: 13.21 mm) compared to men (mean: 13.03 mm), but the difference was not statistically significant. Distal epiphysis width and diaphysis width also showed no significant sex-based differences.

**Table 3:** Anthropometry of the second metacarpal

Measurement (mm)	Sex	Mean	95% Confidence Interval	Variance	Standard Deviation	Minimum	Maximum	Range
Axial Length	F	69.4165	68.5652 - 70.2678	34.441	5.86863	54.67	78.35	23.68
	M	69.3987	68.6126 - 70.1847	34.195	5.84768	55.57	78.38	22.81
Proximal Epiphysis Width	F	13.2074	12.7632 - 13.6515	9.376	3.06199	8.12	21.20	13.08
	M	13.0253	12.6232 - 13.4275	8.95	2.99158	8.22	21.22	13.00
Distal Epiphysis Width	F	10.7436	10.2258 - 11.2615	10.5202	3.23851	10.74	14.10	3.36
	M	10.5424	10.0696 - 11.0151	10.3148	3.22199	10.54	14.86	4.32
Diaphysis Width	F	13.7644	13.3832 - 14.1457	6.907	2.62814	8.78	21.43	12.65
	M	13.7902	13.3952 - 14.1852	8.634	2.93831	8.88	21.34	12.46

Third metacarpal

Table 4 summarizes the measurements for the third metacarpal. Axial length was similar between men (mean: 68.04 mm) and women (mean: 68.06 mm). Proximal epiphysis width was slightly larger in women (mean: 12.16 mm) compared to men (mean: 11.86 mm), but the difference was not statistically significant. Distal epiphysis width and diaphysis width also showed no significant differences.

**Table 4:** Anthropometry of the Third Metacarpal

Measurement (mm)	Sex	Mean	95% Confidence Interval	Variance	Standard Deviation	Minimum	Maximum	Range
Axial Length	F	68.0633	67.2114 - 68.9152	34.491	5.87291	53.71	77.57	23.86
	M	68.0405	67.2580 - 68.8229	33.882	5.82079	54.61	76.67	22.06
Proximal Epiphysis Width	F	12.1585	11.6888 - 12.6283	10.488	3.23851	7.65	20.86	13.21
	M	11.858	11.4249 - 12.2911	10.381	3.22199	7.75	21.63	13.88
Distal Epiphysis Width	F	13.9976	13.5328 - 14.4624	10.268	3.20443	8.00	23.42	15.42
	M	14.1744	13.7193 - 14.6294	11.458	3.38494	9.69	23.33	13.64
Diaphysis Width	F	11.0523	10.5352 - 11.5693	12.705	3.56437	6.77	20.27	13.50
	M	10.7662	10.2966 - 11.2358	12.205	3.49351	6.81	21.16	14.35

Fourth metacarpal

The measurements for the fourth metacarpal are detailed in Table 5. Axial length was nearly identical between men (mean: 61.05 mm) and women (mean: 60.98 mm). Proximal epiphysis width was slightly larger in women (mean: 11.12 mm) compared to men (mean: 10.83 mm), but the difference was not statistically significant. Distal epiphysis width and diaphysis width also showed no significant sex-based differences.

**Table 5:** Anthropometry of the fourth metacarpal

Measurement (mm)	Sex	Mean	95% Confidence Interval	Variance	Standard Deviation	Minimum	Maximum	Range
Axial Length	F	60.9773	60.1484 - 61.8062	32.651	5.71414	49.21	70.36	21.15
	M	61.0469	60.2821 - 61.8117	32.369	5.68938	50.11	70.39	20.28
Proximal Epiphysis Width	F	11.1242	10.5780 - 11.6705	14.182	3.76595	5.21	20.01	14.80
	M	10.8328	10.3188 - 11.3469	14.623	3.82396	5.31	20.80	15.49
Distal Epiphysis Width	F	11.3341	10.9332 - 11.7350	7.639	2.76391	6.78	19.04	12.26
	M	11.5022	11.0995 - 11.9050	11.3634	3.36934	8.98	23.33	14.35
Diaphysis Width	F	8.721	8.2552 - 9.1867	10.31	3.21096	3.75	16.92	13.17
	M	8.5595	8.1295 - 8.9896	10.234	3.19902	3.79	17.14	13.35

Fifth metacarpal

The results for the fifth metacarpal are presented in Table 6. Axial length was similar between men (mean: 53.94 mm) and women (mean: 54.01 mm). Proximal epiphysis width was slightly larger in women (mean: 12.36 mm) compared to men (mean: 12.01 mm), but the difference was not statistically significant. Distal epiphysis width and diaphysis width also showed no significant differences.

**Table 6:** Anthropometry of the fifth metacarpal

Measurement (mm)	Sex	Mean	95% Confidence Interval	Variance	Standard Deviation	Minimum	Maximum	Range
Axial Length	F	54.0125	53.3519 - 54.6731	20.738	4.55393	42.81	64.00	21.19
	M	53.936	53.3305 - 54.5416	20.293	4.50476	43.71	64.03	20.32
Proximal Epiphysis Width	F	12.3623	11.8800 - 12.8446	11.056	3.32500	7.55	20.45	12.90
	M	12.0116	11.5640 - 12.4592	11.086	3.32959	7.65	21.22	13.57
Distal Epiphysis Width	F	11.8716	11.4472 - 12.2960	8.561	2.92588	6.48	20.33	13.85
	M	12.2026	11.7856 - 12.6196	9.624	3.10221	7.23	20.22	12.99
Diaphysis Width	F	10.6832	10.2089 - 11.1574	10.69	3.26954	5.32	17.99	12.67
	M	10.4941	10.0551 - 10.9332	10.667	3.26607	5.42	18.85	13.43

Statistical analysis

Normality testing using the Shapiro-Wilk and Kolmogorov-Smirnov tests indicated that the data did not follow a normal distribution (Table 7). As a result, non-parametric tests (Mann-Whitney U and Wilcoxon W) were used to compare measurements between sexes. The results, presented in Table 8, revealed no statistically significant differences in any of the metacarpal measurements between men and women.

**Table 7:** Normality test for first metacarpus measurements

Variable	Sex	Kolmogorov-Smirnov		Shapiro-Wilk	
		Statistic	p-value	Statistic	p-value
Axial Length	F	0.140	< 0.001	0.934	< 0.001
	M	0.183	< 0.001	0.905	< 0.001
Proximal Epiphysis Width	F	0.145	< 0.001	0.914	< 0.001
	M	0.184	< 0.001	0.884	< 0.001
Distal Epiphysis Width	F	0.120	< 0.001	0.963	< 0.001
	M	0.118	< 0.001	0.934	< 0.001
Diaphysis Width	F	0.139	< 0.001	0.955	< 0.001
	M	0.163	< 0.001	0.921	< 0.001

**Table 8:** Comparison of metacarpal measurements using non-parametric statistical tests

Metacarpal	Measurement	Mann-Whitney U	Wilcoxon W	Z-Score	p-Value
First Metacarpal	Axial Length	18967	42187	-0.798	0.425
	Proximal Epiphysis Width	18649.5	41869.5	-1.074	0.283
	Distal Epiphysis Width	19619	36824	-0.233	0.816
	Diaphysis Width	19755.5	42975.5	-0.114	0.909
Second Metacarpal	Axial Length	19876	37081	-0.01	0.992
	Proximal Epiphysis Width	19090	42310	-0.692	0.489
	Distal Epiphysis Width	18946	42166	-0.817	0.414
	Diaphysis Width	19201.5	42421.5	-0.595	0.552
Third Metacarpal	Axial Length	19781	43001	-0.092	0.926
	Proximal Epiphysis Width	18456	41676	-1.242	0.214
	Distal Epiphysis Width	18925.5	42145.5	-0.834	0.404
	Diaphysis Width	19663	36868	-0.195	0.846
Fourth Metacarpal	Axial Length	19811.5	37016.5	-0.066	0.947
	Proximal Epiphysis Width	18768	41988	-0.971	0.332
	Distal Epiphysis Width	19006	42226	-0.765	0.445
	Diaphysis Width	19748	36953	-0.121	0.904
Fifth Metacarpal	Axial Length	19806	43026	-0.071	0.944
	Proximal Epiphysis Width	18282.5	41502.5	-1.392	0.164
	Distal Epiphysis Width	19029.5	42249.5	-0.744	0.457
	Diaphysis Width	19150	36355	-0.64	0.522

## Discussion

Sex determination in forensic medicine based on the evaluated population is crucial for assessing other parameters of a biological profile, such as height and age. Determining sex from various parts of the body plays a significant role in identifying the profile of an individual under evaluation [17-18]. The metacarpal bones, due to their distinct morphological features and measurable dimensions, have been widely studied for their utility in sex estimation and surgical planning. This study aimed to describe the anthropometric characteristics of metacarpal bones in a Mexican population, providing valuable data for both forensic and clinical applications.

Different studies currently assess the surgical utility of metacarpal measurements. Sephien et al. studied a total of 145 metacarpals from 17 cadavers, concluding that the articular surface can assist in surgical planning [19]. This includes reinforcing structures for screw fixation and contributing to the development of components for joint prostheses. The average measurements support the use of various implant sizes, which depend on both age and sex [19]. In our study, the detailed measurements of metacarpal dimensions provide a foundation for designing implants and prostheses tailored to the Mexican population, particularly in cases of trauma or congenital deformities.

Population-based average estimations have yielded satisfactory results in forensic medicine. For example, a study by Islam et al. on a Bangladeshi population used metacarpal and phalangeal measurements from 292 individuals (145 men and 147 women) without imaging studies [20]. The results were processed through artificial intelligence algorithms to identify sex and age based on average measurements. The algorithms achieved up to 92.5% accuracy when combined with other hypothetical measurements, paving the way for future applications in large-scale forensic bone identification [20]. While our study did not employ artificial intelligence (AI) algorithms, the findings highlight the potential for integrating advanced analytical techniques to enhance the reliability of anthropometric analyses in the Mexican population.

Our study stands out in the national literature for including the largest number of patients and the most comprehensive set of measurements from patients in a hospital setting. In comparison, Torres et al. conducted a study on metacarpal measurements in a Mexican population, reporting substantial differences between sexes. Their study found that men had larger diameters in all measurements; however, their sample consisted of skeletons from unclaimed bodies, whereas our study utilized standardized radiographic measurements [21]. This methodological difference may account for the discrepancies in findings, as radiographic measurements can be influenced by factors such as soft tissue thickness and imaging quality.

Study limitations

Even though our study accurately classified the patients, there is some limitations to be considered, first, as the sample was derived from people living in Mexico City, this is a minority in the total population of our country. This may limit the generalization of findings to the wider national population in forensic contexts. The second point is that the use of 2D radiographs, though practical and widely accessible, may not capture the full complexity of bone morphology compared to 3D imaging modalities like CT or MRI, which might further contextualize the findings. Advanced imaging techniques could provide more detailed and accurate measurements, possibly revealing subtle differences between sexes.

Moreover, the research did not control for other factors that may impact metacarpal dimensions, such as age, height, or genetic influences. These should also be included in further studies to strengthen analyses. Lastly, the relatively small sample size, with 400 cases, may have lowered the statistical power and reduced the likelihood of observing significant differences between sexes. The lack of significant sex differences may reflect population-specific homogeneity, limitations of 2D radiography, or the influence of unmeasured confounders.

Clinical and forensic Implications

Despite these limitations, this study represents one of the most extensive investigations of metacarpal dimensions in a hospital-based Mexican population. The findings provide a foundation for future research and have important implications for both clinical and forensic applications. Accurate knowledge of metacarpal dimensions is essential for surgical planning in clinical settings, particularly in trauma or congenital deformities. The data from this study can inform the design of implants and prostheses tailored to the Mexican population.

In forensic medicine, the lack of significant sex-based differences in metacarpal dimensions suggests that additional skeletal elements or advanced analytical techniques may be required for accurate sex estimation in the Mexican population. Future studies should explore the use of machine learning algorithms or artificial intelligence to analyze large datasets and identify subtle patterns that may not be apparent through traditional statistical methods.

Future directions

Future studies should, therefore, be designed to include measurements of other hand bones for a more comprehensive understanding of sex-based differences. Advanced imaging modalities, such as CT or MRI, should be employed to improve the accuracy and reliability of measurements. Additionally, machine learning algorithms could be used to analyze and predict sex determination more effectively. Works like Islam et al. showcase the potential of AI in enhancing the reliability of anthropometric analyses [20]. Applying such methods to larger datasets in the Mexican population could yield valuable insights and practical forensic applications.

## Conclusions

The study revealed distinct mean values for metacarpal measurements between men and women, with women generally exhibiting larger measurements across most dimensions. However, statistical tests, including the Shapiro-Wilk and Kolmogorov-Smirnov tests, indicated that the data did not follow a normal distribution. Consequently, non-parametric tests such as the Mann-Whitney U and Wilcoxon W were employed, which did not identify statistically significant differences between the sexes.

These findings highlight the potential utility of metacarpal measurements in forensic and surgical contexts, particularly in population-specific studies. However, the lack of significant differences between sexes suggests that metacarpal dimensions alone may not be sufficient for sex determination in forensic cases. Future research should incorporate additional imaging modalities, such as CT or MRI, to capture more detailed morphological data. Furthermore, expanding the study to include measurements from other hand bones and utilizing advanced analytical techniques, such as machine learning algorithms, could enhance the reliability and applicability of these findings.

## References

[1] Muñoz García (2025). The application of osteometry in human identification: the estimation of sex and ancestry in the contemporary Spanish population. Universidad Complutense de Madrid.

[2] Torres-Ramírez G (2025). Study of the metacarpus and metatarsus for sex assignment in the contemporary Mexican human population. Universidad Nacional Autónoma de México (ed): Universidad Nacional Autónoma de México, México.

[3] Tountas CP, Bergman RA (1993). Anatomic variations of the upper extremity. Anatomic Variations ofthe Upper Extremity, pp. 196±210.. Churchill-Livingstone, New York.

[4] Barrio PA, Trancho GJ, Sánchez JA (2006). Metacarpal sexual determination in a Spanish population. J Forensic Sci.

[5] El Morsi DA, Al Hawary AA (2013). Sex determination by the length of metacarpals and phalanges: X-ray study on Egyptian population. J Forensic Leg Med.

[6] Scheuer L (2002). Application of osteology to forensic medicine. Clin Anat.

[7] Testut L, Latarjet A (1969). Treatise on Human Anatomy (A book in Spanish). Salvat (ed.

[8] Dalley A, Moore KL, Agur AM (2002). Clinically oriented anatomy. F: Clinically oriented anatomy . Panamericana (ed): Panamericana, Buenos Aires.

[9] Williams PL, Warwick R, Dyson M, Bannister L (1989). Gray's Anatomy, 37th Edition. Gray Anatomy..

[10] Eshak GA, Ahmed HM, Abdel Gawad EA (2011). Gender determination from hand bones length and volume using multidetector computed tomography: a study in Egyptian people. J Forensic Leg Med.

[11] Khanpetch P, Prasitwattanseree S, Case DT, Mahakkanukrauh P (2012). Determination of sex from the metacarpals in a Thai population. Forensic Sci Int.

[12] Scheuer JL, Elkington NM (1993). Sex determination from metacarpals and the first proximal phalanx. J Forensic Sci.

[13] Case DT, Rawlins CM, Mick CB (2015). Measurement standards for human metacarpals. Am J Phys Anthropol.

[14] Mohamed MM, Ahmed HM, Hassan OA (2021). Reliability of internal metacarpal measurements for sex determination using multi-detector computed tomographic imaging in a sample of Egyptian population. Aus J Forensic Sci.

[15] (2025). Synapse Mobility Login imaging viewer. https://healthcaresolutions-us.fujifilm.com/products/enterprise-imaging/synapse-mobility-enterprise-viewer/.

[16] (2025). IBM SPSS Statistics for Windows. https://www.ibm.com/support/pages/downloading-ibm-spss-statistics-25.

[17] Hemy N, Flavel A, Ishak NI, Franklin D (2013). Sex estimation using anthropometry of feet and footprints in a Western Australian population. Forensic Sci Int.

[18] Abdel Fatah E, Shirley SR, Jantz RL, Mahfouz MR (2014). Improving sex estimation from crania using anovel three-dimensional quantitative method. J Forensic Sci.

[19] Sephien A, Bethel CF, Gulick D, Nairn C, Ourn F, Schwartz-Fernandes FA (2021). Inter-relationships of metacarpals 1 to 5, regarding their length, metaphyseal midshaft width, articular surface area of head and base, age, and sex: a cadaveric study. Hand (N Y).

[20] Islam MZ, Supto MR, Asadujjaman M, Chakrabortty RK (2021). Sex identification from hand measurements using machine learning. International Conference on Computing, Communication, and Intelligent Systems (ICCCIS).

[21] Torres G, Garmendia AM, Sánchez-Mejorada G, Gomez-Valdes JA (2020). Estimation of gender from metacarpals and metatarsals in a Mexican population. Revista Española de Medicina Legal.

